# Fatigue Properties and Its Prediction of Polymer Concrete for the Repair of Asphalt Pavements

**DOI:** 10.3390/polym14142941

**Published:** 2022-07-20

**Authors:** Senzhi Ren, Xin Hu

**Affiliations:** School of Civil Engineering, Central South University of Forestry and Technology, Changsha 410004, China; rensenzhi@126.com

**Keywords:** polymer concrete, fatigue property, semi-circular bending test, stress level

## Abstract

Polymer concrete (PC) is considered a promising repair material for asphalt pavement, since it has excellent paving performance and water stability. Although the mechanical properties of PC have been widely researched, the fatigue behavior of PC under traffic loads was still poorly understood. To predict the fatigue life and optimize the material design of PC, the semi-circular bending (SCB) tests were performed, considering different polymer content, sand ratio, aggregate features and stress condition. Two typical polymer materials were applied to prepare PC specimens, including epoxy resin (ER) and polyurethane (PU). The aggregate features were analyzed by the aggregate image measurement system. The mechanical behavior under repeated loads was investigated by the displacement, fatigue life and stiffness modulus. Results show that the flexural strength increases nonlinearly with the increasing polymer content, rapidly at first, and then slowly. The optimized polymer content and sand ratio were respectively 15% and 30%. As the loading number increases, the vertical displacement of PC shows three stages, i.e., undamaged stage, damage development stage, and fatigue failure stage. The stiffness modulus of the specimen is stress-dependent. An empirical model was developed to predict the fatigue life of PC, which can effectively capture the effects of the polymer content, sand ratio and stress level (or nominal stress ratio). It suggests that the fatigue life has a strong correlation with the mixing gradation, and the optimal sand ratio of PC can be determined by the proposed function. Moreover, the effect of aggregate shapes cannot be neglected.

## 1. Introduction

As the main form of pavement structure in China, asphalt pavement plays an important role in the service performance of highways [1]. Under the long-term combined actions of the environment and loads, including heat, aging, rainfall, and repeated loading, asphalt pavement may develop diseases such as pit slots and cracks [2]. These diseases damage the stability of the pavement structure and reduce its operational efficiency. Statistical analysis shows that China’s annual asphalt pavement maintenance mileage is over 100,000 km, and the cost of pavement repair alone is up to 2.3 billion yuan [3]. As the traffic volume and quality requirements of highways continue to increase, a fast and effective treatment method is required for accelerated repair and reopening of damaged highways. In general, pavement repair is conducted regardless of weather conditions. Hot mix asphalt is the most common repair material, yet it requires high-temperature heating and is not environmentally friendly [4]. In the meantime, the bonding between old and fresh asphalt mixtures is relatively weaker, and the penetration depth of fresh asphalt is low in small cracks, which limits its application in pavement repair [5]. As a supplement, various special polymer materials, including emulsified asphalt [6], polyurethane (PU) [7], and epoxy resin (ER) [8] have been adopted in pavement engineering. Polymer materials exhibit several excellent performances in terms of the corrosion resistance [9], inflaming retarding [10], thermal stability [11], and fracture toughness [12], etc. These new features were granted to asphalt pavement by modifying or mixing with corresponding polymers.

Polymer concrete (PC), a composite material consisting of various types of aggregates and a polymer matrix, is considered a promising material for pavement repair due to its fast curing and strong bonding with aggregates [13]. Compared with traditional asphalt mixtures, it has higher chemical resistance, better high-temperature stability, and good durability in field environments [14]. PU is a common polymeric binder that has been widely used in materials for drainage pavement structures, such as porous PU mixtures [15]. Adding fillers or changing the polymeric structure could improve the UV resistance, aging resistance, water stability, and freeze-thaw resistance of PU-based materials [16,17]. Ma, et al. [18] found through laboratory tests and field application that the high-temperature performance, low-temperature performance, deformation coordination performance, and fatigue resistance of PU–PC were better than cast asphalt concrete and epoxy asphalt concrete. Various studies have been conducted on the mechanical properties and reinforcement techniques of PC [19,20]. For example, the pavement performance of PC, such as compressive strength, fracture toughness, and flexural stiffness, has been studied through laboratory tests and numerical analysis [21]. In terms of material development, various composited and modified PC designs and blending solutions, such as fiber-reinforced PC [22] and ER/PU polymer concrete [23], have been investigated to achieve various performance enhancements. Test methods for the interface shear strength have also been developed to evaluate the bonding properties between PC and asphalt mixtures [24]. These studies have greatly improved the understanding of the mechanical properties of PC.

Fatigue cracking, a common disease in asphalt pavements, is one of the main considerations in their structural design [25]. However, the existing literature somehow neglected the fatigue properties of PC as a repair material of pavement [26]. Zaghloul, et al. [27,28] found both the stress level and the fiber content have a great influence on the tensile and fatigue behaviors of fiber-reinforced polyester. Yeon, et al. [29] and Ahn, et al. [30] investigated the fatigue performance of PC and its stiffness damage through bending beam tests and vibration measurements, but mainly focused on the effects of test methods on the fatigue characteristics of PC. The effects of component types and contents on fatigue performance were not fully revealed in previous research. Meanwhile, considering the effects of vehicle loading and temperature, the fatigue performance of PC under different conditions should be studied, since its damage mechanism is different from that under the static loading [31,32]. Due to the large number of trials and the complex processes, the construction sites lack the conditions for the required tests. Therefore, it is necessary to establish a PC fatigue life prediction equation to facilitate the performance evaluation and maintenance timing of this repair material [33]. Moreover, existing studies have not simultaneously considered the effects of polymer content, aggregate gradation, shape characteristics, and stress condition on the fatigue life of PC. The optimal design of PC lacks the sufficient data about dynamic tests.

In this study, PCs with ER and PU binders were prepared, respectively, for the rapid repair of asphalt pavements. Semi-circular bending (SCB) tests were carried out to analyze the effects of different polymer contents, aggregate characteristics, and stress levels on the mechanical properties of PC. Indexes such as displacement, fatigue life, and the stiffness modulus were used to evaluate fatigue performance. Finally, a PC fatigue life prediction equation considering the combined effect of several factors was established.

## 2. Materials and Methods

### 2.1. Materials

#### 2.1.1. Aggregates

**Figure 1:**
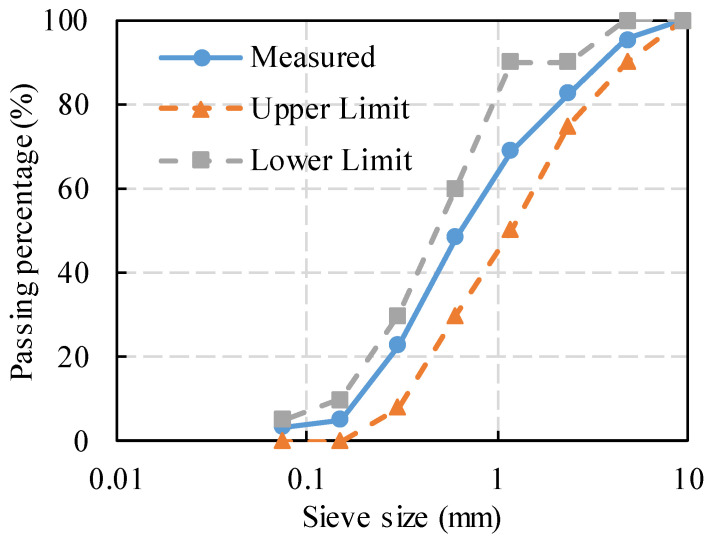
Result of sand screening test.

The fine aggregate used in this test was the natural sand produced from a quarry at Zhuzhou, China, which had a fineness modulus of 2.8 and a mud content below 1.0%. Its screen test result is shown in Figure 1. The coarse aggregate used in the tests was the continuously graded basalt gravel with a size of 5–16 mm. Table 1 shows the mechanical properties of the coarse aggregate, which conforms to the Chinese Standard for Technical Requirements and Test Method of Sand and Crushed Stone (or Gravel) for Ordinary Concrete (JGJ 52-2006).

#### 2.1.2. Polymer Binders

The polymer binders used in this study were ER and PU. The ER binder had a longer initial setting time and greater viscosity than typical PU materials. Meanwhile, the initial viscosity and setting time of the polymer matrix were controllable [34]. ER was prepared with the E51 ER, the triethylenetetramine curing agent, and the DMP-30 accelerator (100:9:1). The synthetic materials of PU mainly included isocyanate monomer and polyether. To ensure enough time for paving and compacting in the road repair project, the 6170F single-component PU (Wanhua Chemical Company, Yantai, China) was selected as the PU binder. The test results of polymer binders are shown in Table 2.

### 2.2. Sample Preparation

**Figure 2:**
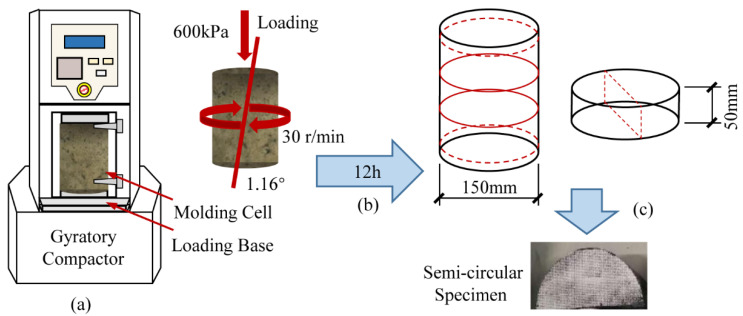
Preparation process of semicircular specimens: (**a**) compaction; (**b**) curing for 12 h; (**c**) cutting into semi-circular halves.

The PC specimens were prepared in the laboratory by mixing the aggregates and polymer binders according to the ASTM C192 standard. The mixing ratios of aggregates to polymer matrix were 95:5, 90:10, 85:15, and 80:20 by weight. The sand ratios were 25%, 30%, and 35%, respectively. The well-mixed PC was poured into test molds pre-applied with a release agent. Cylindrical specimens 150 mm in diameter and 180 mm in height were molded by the Superpave rotary compactor. During compaction, the compressive stress was 600 kPa, the offset angle was 1.16°, and the rotation rate was 30 r/min. Each prepared specimen was compacted 100 times to ensure uniform mass density distribution. The specimens were demolded 1 h after molding. Before use, the final specimen was conditioned at room (25 °C and a standard atmospheric pressure) for 12 h [35]. Wang, et al. [36] found that the internal stress of specimens above 50 mm in thickness tends to planar strain state, and the stress response tends to stabilize. Therefore, the specimens for SCB tests were cut into semi-circular halves with a diameter of 150 mm and a thickness of 50 mm. The preparation process of the SCB specimens is shown in Figure 2.

### 2.3. Testing Methods

#### 2.3.1. Aggregate Feature Measurement

The morphology characteristics of the coarse aggregates were analyzed by the aggregate image measurement system (AIMS). Three different shape indicators of the aggregates were measured, namely: surface texture, gradient angularity, and sphericity. The surface texture was described through wavelet analysis based on transverse, longitudinal, and oblique images of the tested aggregates. Angularity was measured using the gradient approach to quantify the variation in size and profile of the aggregate particles. The three-dimensional shape characteristics were evaluated by sphericity, which was calculated by the long axis length, the middle axis length, and the short axis length of the aggregate bounding box. The sphericity ranges from 0 to 1, and when it approaches 1, the measured aggregate is approximately spherical in shape. The detailed descriptions can be found in the previous literature [37].

Table 3 shows the measured results of coarse aggregates with a diameter of 5–16 mm. The results show that the angularity distribution of total aggregates meets the requirements of pavement performance due to the small content of flat and elongated particles. AIMS tests revealed relatively pronounced differences in the quantitative indicators of the aggregates, such as texture and angularity, and their effects on the performance of the specimens need further investigation.

#### 2.3.2. Semi-Circular Bending Test

The SCB tests were conducted using the dynamic universal testing machine UTM-100, composed of the constant temperature incubator, loading platform, and servo controller. The selected loading mode was the three-point bending method. The specimens were set on beam support with a loading roller above and two support rollers beneath, all of which were 1 mm in diameter. The distance between the two support rollers was set to 0.8 times the specimen diameter. Before testing, a contact load of 0.2 kN was applied and held for 10 s to ensure uniform contact between the specimen and the loading roller. The test temperature was set to 15 °C, according to the annual temperature gradient variation of Hunan Province. A constant loading rate of 50 mm/min was maintained in the SCB strength tests until the cracking failure of the specimen. The tests stopped as the load dropped to 0.3 kN. The tensile strength of specimens can be determined by SCB tests with single loading. The maximum tensile stress at the bottom of the SCB specimen can be calculated with Equation (1). For the SCB fatigue tests, the loading was applied in the form of haversine waves at a frequency of 10 Hz. The stress levels were set at four different loading stress ratios (0.2, 0.3, 0.4 and 0.5), based on the SCB strength. For each stress level, three parallel tests were performed to ensure the reliability of the fatigue tests. The testing results in the figures represent an average result by parallel tests.

(1) $$\sigma_{t}=\frac{4.976P}{TD}$$

where, *σ_t_* stands for the maximum tensile stress at the bottom of SCB specimen; *P* is the vertical loading; *T* and *D* are respectively the thickness and semicircle diameter of the SCB specimen.

In the SCB fatigue tests, unrecoverable damage occurred inside the PC specimens as the number of loadings increased, which led to the mechanical property decay of the material. The effect of fatigue damage on the mechanical properties can be described by the decay of the stiffness modulus. The stiffness modulus is the stress to strain ratio at the bottom center of the SCB specimen in tension, as presented in Equation (2). Assuming that the stress distribution in the span section of the SCB specimen conforms to the flat section assumption, the tensile strain at the bottom center of the specimen can be expressed as Equation (3).

(2) $$S_{t}=\frac{\sigma_{t}}{\varepsilon_{t}}$$

(3) $$\varepsilon_{t}=\frac{6Ld}{1.14D^{2}\left(5.578\frac{L}{D}-1.3697\right)}$$

where, *S_t_* stands for the stiffness modulus; *ε_t_* stands for the tensile strain at the center of the bottom of the SCB specimen; *L* is the distance between adjacent fixing supports; *d* is the deflection at the center of the SCB specimen.

## 3. Results

### 3.1. SCB Strength Test

Table 4 shows the SCB strength test results under the three different mixing proportions and different polymer contents. The polymer content represents the percentage of polymer binder in the PC by weight. The flexural strength of PC at different sand ratios varies significantly with polymer content. Overall, the flexural strength increases nonlinearly with the increasing polymer content, rapidly at first and then slowly. The main reason is that at low polymer content, the binder cannot completely coat the aggregate surface to develop sufficient interfacial strength. In this study, the flexural strength of PC was less affected by the sand ratio. When the sand ratio is 30%, the bending strengths of ER–PC and PU–PC peaked at 18.82 MPa and 15.24 MPa, respectively. The strength of ER–PC is higher than that of PU–PC, which is consistent with the physical test results of the polymers. As the sand ratio exceeds 30%, the flexural strength of the PC decreases. The main reason is that the excessively low sand ratio causes incompletely filled voids between coarse aggregates. At the same time, an excessive sand ratio reduces the amount of coarse aggregate and increases the total surface area of coarse and fine aggregates, leading to an increased amount of PU. As shown in Table 4, the variability of the strength test was small, with all coefficients of variation (CV) in the parallel tests below 10%. Therefore, considering the economy and design strength requirements, the sand ratio for the tests was determined to be 30%, and the initial admixture of the polymer was 15%.

### 3.2. Displacement Changes of SCB Fatigue Test

**Figure 3:**
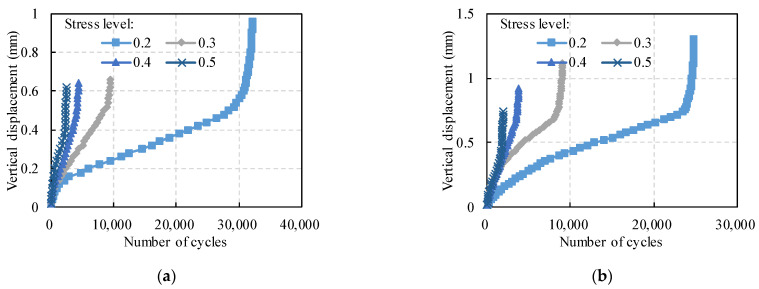
Displacement changes of SCB fatigue test: (**a**) ER–PC; (**b**) PU–PC.

The fatigue damage process is as follows. Under repeated loading, the asphalt mixture gradually sustains plastic deformation and cumulative damage, finally leading to fracture damage. The plastic deformation trend of the PC was analyzed with SCB fatigue tests. Taking the 30% sand ratio and the 15% polymer content as an example, Figure 3 shows the displacement of the two kinds of PCs under different stress levels.

As the number of cycles increases, the vertical displacement curve shows three stages, i.e., undamaged stage, damage development stage, and fatigue failure stage. In the initial stage of loading, the specimen undergoes elastic-plastic deformation due to internal contact and void compression [38]. The permanent deformation increases significantly with the increasing number of loadings. At the second stage of loading, the vertical displacement increases linearly with the number of loadings. This time, microcracks appear and develop steadily inside the specimen. In the third stage, an inflection point appears on the loading curve, and the specimen fails. Elseifi, et al. [39] found that the microdamage concentrated mainly in the middle of the specimen and then produced penetration cracks. The criterion for fatigue damage is usually defined as the critical point between the damage development stage and the fatigue failure stage. Figure 3 shows that as the stress level increases, the fatigue life and final displacement of the specimen tend to decrease. The fatigue life of ER–PC is greater than that of PU–PC in this study.

### 3.3. Attenuation of Stiffness Modulus with Loading Cycles

**Figure 4:**
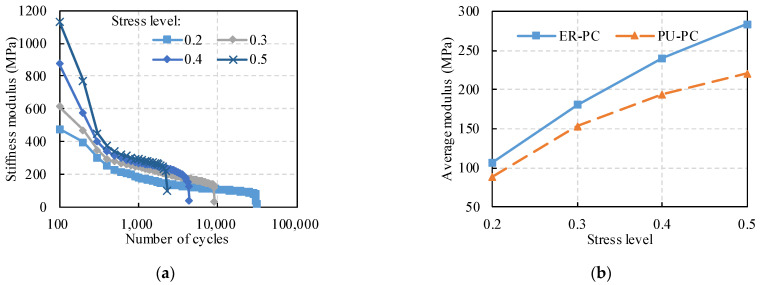
Stiffness modulus of SCB fatigue test: (**a**) changes of ER–PC with cycle number; (**b**) Average modulus of ER–PC and PU–PC.

Figure 4a shows that the stiffness modulus changes in the SCB tests under different stress levels. The sand ratio of the specimens is 30%, and the polymer content is 15%. It is obvious that the stiffness modulus of the PC decreased continuously during the tests, indicating that the reduced part of the stiffness modulus is attributed to the fatigue damage of the specimens [40]. In the damage development stage, the elastic deformation of PC can fully recover after unloading, but the damage deformation it produces is not recoverable. As a result, the stiffness modulus of PC decays, and damage deformation occurs.

The variation trends of the stiffness modulus can be divided into three stages. In the first stage, cracking occurs at the surface and internal defects of the specimen, such as cracks and voids, resulting in a sharp decrease in the stiffness modulus due to stress concentration. In the second stage, as the number of loadings increases, the high-density energy at the internal defects of the specimen is released due to the cracking. Meanwhile, the fatigue damage development is inhibited, manifested as the linear decrease in the stiffness modulus. In the third stage, the stiffness modulus of the asphalt mixture decreases sharply, manifesting as severe damage and rapidly increasing fatigue damage rate until failure.

In the damage development stage, the average stiffness modulus is displayed in Figure 4b to compare the elastic properties of the two PC. The results show that the stiffness modulus of the specimen is dependent on the stress condition. The average modulus of the specimens increases approximately linearly with the increasing stress. Due to the better adhesion between ER and aggregate, the stiffness modulus of ER–PC is larger than that of PU–PC.

### 3.4. Evaluation and Prediction of Fatigue Life

**Figure 5:**
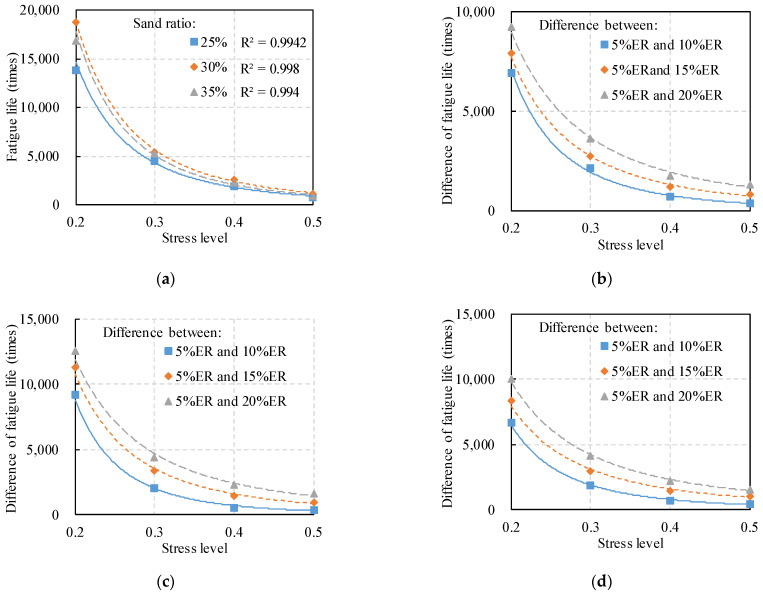
Effect of stress level on the fatigue life of ER–PC: (**a**) 5% ER content; (**b**) sand ratio of 25%; (**c**) sand ratio of 30%; (**d**) sand ratio of 35%.

The above analysis reveals that the fatigue performance of the PC specimens is mainly related to three factors, i.e., sand ratio, polymer content, and stress level. Additionally, the morphological index of the aggregates was introduced in this study for fatigue life prediction. The fatigue test results are presented in Table 5. The framework of the fatigue prediction equation is established in the following steps. Firstly, the effect of stress level is considered, and the standard *σ*-*N* fatigue equation is adopted for predicting the fatigue life, which can be expressed as Equation (4). Taking 5% ER content as an example, the relationship between the fatigue life and the stress level were decided by multivariate nonlinear regression analysis, according to Equation (4). Figure 5a shows the fatigue prediction results of PC specimens with different sand ratios. Table 5 presents all predicted results of *σ*-*N* fatigue equation. The fatigue life showed a good correlation with stress levels, since the determination coefficients of Equation (4) is higher than 0.99.

(4) $$N_{f}\left(\sigma \right)=K_{1}{\left(\frac{1}{\sigma }\right)}^{K_{2}}$$

where, *N_f_* stands for the fatigue life; *σ* is the stress level; *K*_1_ and *K*_2_ is the fitting parameters.

Secondly, the fatigue life difference of PC specimens with different ER contents has a similar power function relationship with the stress level, as shown in Figure 5b–d. Therefore, the function of fatigue life difference with the fatigue life of the 5% ER–PC as the reference can be expressed as Equation (5).

(5) $$\Delta N_{f}\left(\sigma ,\Delta c_{p},S_{r}\right)=K_{3}\left(\Delta c_{p},S_{r}\right){\left(\frac{1}{\sigma }\right)}^{K_{4}\left(\Delta c_{p},S_{r}\right)}$$

where, Δ*c_p_* stands for the relative content of polymer binder; *S_r_* stands for sand ratio; *K*_3_ and *K*_4_ are the model parameters considering polymer content and sand ratio.

Thus, the fatigue life of PC specimens with different ER contents can be determined by Equation (6). Moreover, the basic frame of fatigue life prediction is still a power function, since the *K*_3_ is greater than 0. It implies that the stress level plays an essential role for the predictability and accuracy of the proposed fatigue function.

(6) $$\begin{array}{ll} N_{f}\left(\sigma ,\Delta c_{p},S_{r}\right) & =N_{f}\left(\sigma ,0,S_{r}\right)+\Delta N_{f}\left(\sigma ,\Delta c_{p},S_{r}\right)\\ & =K_{1}{\left(\frac{1}{\sigma }\right)}^{K_{2}}+K_{3}\left(\Delta c_{p},S_{r}\right){\left(\frac{1}{\sigma }\right)}^{K_{4}\left(\Delta c_{p},S_{r}\right)} \end{array}$$

**Figure 6:**
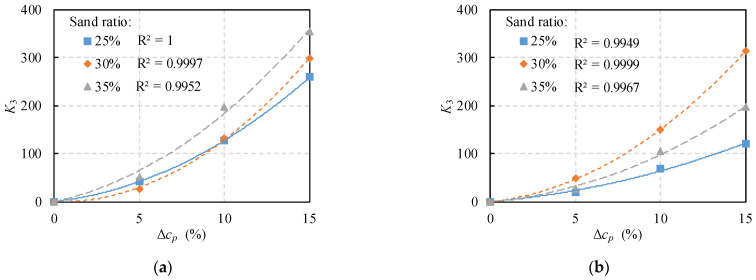
Fitting results of coefficient *K*_3_ with different Δ*c_p_*: (**a**) ER–PC; (**b**) PU–PC.

In the case of the same sand ratio, the relationship between the coefficient *K*_3_ and the relative polymer content was fitted in Figure 6. The fatigue life difference should be 0 at the ER content of 5% (Δ*c_p_* = 0). Thus, the y-intercept of the fatigue life difference function is set to 0. Meanwhile, to ensure the accuracy of the prediction model, the determination coefficient of the *K*_3_ fitting results should be above 98%. Therefore, the fitted model takes the form of a quadratic function, as expressed in Equation (7). To verify the reliability of Equation (7), Table 6 shows the fitting results of ER–PC and PU–PC. The results suggest that the fitted equations are applicable to predict the fatigue life of both materials.

(7) $$K_{3}\left(\Delta c_{p}\right)=a{\left(\Delta c_{p}\right)}^{2}+b\Delta c_{p}$$

where, *a* and *b* are the fitting parameters.

Thirdly, coefficients *K*_1_ and *K*_2_ of the fatigue equation are analyzed. The gradation (or sand ratio) of the mixture significantly contributes to the mechanical properties of PC. The optimized gradation is often determined by static mechanical tests, such as compaction tests or uniaxial compression tests. These static design methods are unreasonable since the real pavement response is under dynamic traffic loading. However, previous literature rarely mentioned the mixing design method considering the fatigue life. This study supplied a novel view about the design of PC.

Table 7 presents the details of material properties and predicted parameters, taking 5% polymer content as an example. Results shows that there is no definitive link between the sand ratio and the coefficient *K*_2_. Nevertheless, the coefficient *K*_1_ shows a strong correlation with the type of binder and the sand ratio. According to the results of Table 6, the empirical relationship between tensile strength, sand ratio, and coefficient *K*_1_ was established by multivariate nonlinear regression, as expressed in Equation (8). For other polymer contents in this study, the results show similar functional relationships. It suggests that the fatigue life of the specimen is proportional to the tensile strength of the binder, and a suitable gradation could optimize the performance of the PC. Especially, in this case of 5% polymer content, the optimal sand ratio of PC should be 30.237%.

(8) $$K_{1}\left(T_{s},S_{r}\right)=T_{s}\left[-0.674{\left(S_{r}-30.237\right)}^{2}+60.221\right],\;R^{2}=96.95\%$$

**Figure 7:**
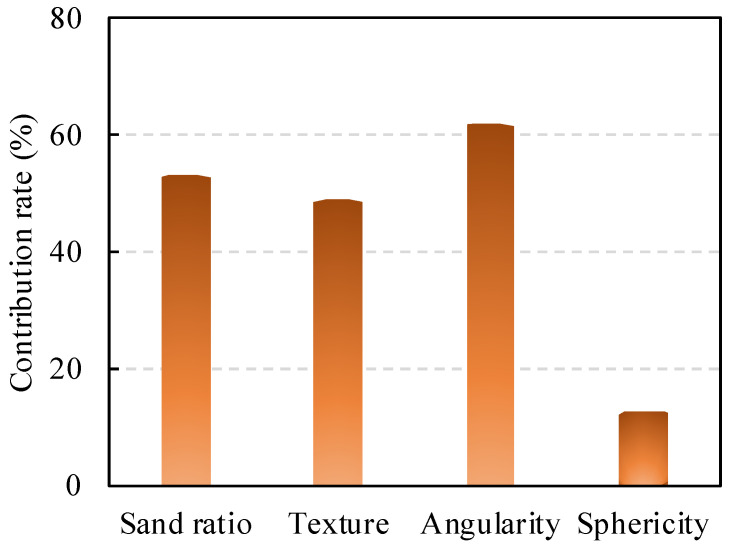
Contribution rate of material properties on *K*_2_.

Furthermore, the fatigue performance of PC is affected by the interlocking effect of the aggregate structure and the interfacial properties [41]. Through experiments and numerical analysis [37], Yao, et al. [42] found that the shape characteristics of aggregate are related to the stress dependence of deformation behavior. The univariate predictor variables of coefficient *K*_2_ were screened using the Bootstrap Forest model in Figure 7. It is proved that the angularity of aggregates has the strongest correlation with *K*_2_, followed by the sand ratio and texture, whereas sphericity has the weakest correlation with *K*_2_.

## 4. Conclusions

This study carried out the SCB tests of PC considering the polymer content, aggregate shape and stress level. The main finding can be drawn as follow:

(1) Based on the SCB strength test results, it shows that the polymer content and sand ratio have significant influence on the flexural strength. The strength increases nonlinearly with the increasing polymer content, rapidly at first and then slowly. However, as the sand ratio exceeds 30%, the flexural strength of the PC decreases.
(2) According to displacement changes of PC under repeated loadings, the testing process presents three stages, i.e., undamaged stage, damage development stage, and fatigue failure stage, as the number of cycles increases. Moreover, the stress level increases, and the fatigue life and final displacement tend to decrease.
(3) In terms of the stiffness modulus, the fatigue damage of specimens may result in the decay of the stiffness modulus. Meanwhile, the stiffness modulus is dependent on the stress level. The average modulus of the specimens increases approximately linearly with the increasing stress.
(4) A prediction model of fatigue life is established containing stress level, polymer content, tensile strength and sand ratio. The basic frame of fatigue life prediction is a power function, and the stress level plays an essential role for the predictability and accuracy.
(5) The fatigue life has a strong correlation with the type of binder and the mixing gradation. Meanwhile, the optimal sand ratio of PC can be determined by the proposed empirical function. According to aggregate shape analysis, the effects of angularity and texture on fatigue life are more significant, whereas the effect of sphericity is relatively weak.

## Figures and Tables

**Table 1:** Mechanical properties of coarse aggregates.

**Items**	**Units**	**Results**		**Requirements**
		5–10 mm	10–16 mm	
Apparent density	g/cm^3^	2.718	2.735	≥2.65
Crushing value	%	16.7	17.4	≤22
Water absorption	%	0.43	0.41	≤1.5
Los Angeles attrition loss	%	16.0	16.7	≤22.0
Mud content	%	0.45	0.3	≤0.8

**Table 2:** Physical and chemical properties of polymer binders.

Items	Units	Epoxy Resin	Polyurethane
Density	g/cm^3^	1.317	1.117
PH	—	7.5	8.1
Melting point	°C	252	175
Thermal expansion	μm/mK	54	160
Viscosity	MPa·s, 25 °C	2734	1233
Tensile strength	MPa	2.7	1.9
Elongation at break	%	200	550
Curing time	h	12	≤12

**Table 3:** Morphology measurement of basalt aggregates.

Particle Size (mm)	Value	Percentage of Flat- elongated Particles (%)	Texture	Gradient Angularity	Sphericity
5–10	Mean	8.34	343.6	3283	0.67
	Standard deviation	—	93.5	802.5	0.08
10–16	Mean	7.98	422.9	2961	0.69
	Standard deviation	—	124.2	671.8	0.09

**Table 4:** SCB strength with different sand ratio and polymer content.

Sand Ratio (%)	Polymer Content (%)	ER–PC		PU–PC	
		SCB Strength (MPa)	CV(%)	SCB Strength (MPa)	CV(%)
25	5	2.29	5.80	1.28	4.17
	10	9.88	5.40	7.34	4.29
	15	14.05	5.31	11.97	5.09
	20	16.68	5.91	13.51	4.22
30	5	2.98	4.92	1.76	4.47
	10	12.02	5.76	5.94	4.15
	15	16.27	4.30	13.19	4.43
	20	18.82	6.09	15.24	5.10
35	5	2.71	4.55	1.49	4.62
	10	10.63	5.81	4.91	4.19
	15	15.11	5.19	11.27	4.82
	20	17.83	6.44	14.45	4.83

**Table 5:** Fitting results of *σ*-*N* fatigue equation.

Sand Ratio (%)	Polymer Content (%)	ER–PC			PU–PC		
		*K*_1_	*K*_2_	*R*^2^ (%)	*K*_1_	*K*_2_	*R*^2^ (%)
25	5	110.910	3.033	99.42	81.855	3.005	99.05
	10	152.310	3.082	99.77	97.822	3.134	99.58
	15	235.350	2.824	99.95	150.340	2.883	99.77
	20	343.560	2.618	99.99	200.460	2.758	99.91
30	5	157.050	2.972	99.8	122.080	3.079	99.62
	10	176.190	3.141	99.98	170.620	3.114	99.86
	15	289.830	2.872	99.95	265.170	2.887	99.94
	20	431.950	2.643	99.85	398.970	2.657	99.93
35	5	119.320	3.109	99.4	88.262	3.083	99.6
	10	172.020	3.074	99.85	112.890	3.171	99.95
	15	296.260	2.763	99.99	189.090	2.879	99.95
	20	415.400	2.593	99.99	265.690	2.691	99.98

**Table 6:** SCB strength with different sand ratio and content.

Materials	Sand Ratio (%)	*a*	*b*	*R*^2^ (%)
ER–PC	25	0.8959	3.8454	1
	30	1.4134	−1.1837	99.97
	35	1.1071	7.3932	99.54
PU–PC	25	0.3479	2.9345	99.49
	30	1.1604	3.5212	99.99
	35	0.6810	3.0317	99.67

**Table 7:** Relationships between materials properties and predicted parameters.

Polymer Matrix	Tensile Strength (MPa)	Sand Ratio (%)	Aggregate Morphology			*K*_1_	*K*_2_
			Texture	Angularity	Sphericity		
ER	2.7	25	368.2	3056	0.69	110.91	3.033
	2.7	30	359.2	2991	0.69	157.05	2.972
	2.7	35	368.5	3263	0.68	119.32	3.109
PU	1.9	25	365.6	2967	0.67	81.855	3.006
	1.9	30	368.4	3197	0.68	122.08	3.079
	1.9	35	372.2	3270	0.68	88.262	3.083

## Data Availability

Not applicable.
